# Weighted Synapses Without Carry Operations for RRAM-Based Neuromorphic Systems

**DOI:** 10.3389/fnins.2018.00167

**Published:** 2018-03-16

**Authors:** Yan Liao, Ning Deng, Huaqiang Wu, Bin Gao, Qingtian Zhang, He Qian

**Affiliations:** Institute of Microelectronics, Tsinghua University, Beijing, China

**Keywords:** weighted synapses, neural networks, sign stochastic gradient descent, online learning, resistive random-access memory (RRAM)

## Abstract

The parallel updating scheme of RRAM-based analog neuromorphic systems based on sign stochastic gradient descent (SGD) can dramatically accelerate the training of neural networks. However, sign SGD can decrease accuracy. Also, some non-ideal factors of RRAM devices, such as intrinsic variations and the quantity of intermediate states, may significantly damage their convergence. In this paper, we analyzed the effects of these issues on the parallel updating scheme and found that it performed poorly on the task of MNIST recognition when the number of intermediate states was limited or the variation was too large. Thus, we propose a weighted synapse method to optimize the parallel updating scheme. Weighted synapses consist of major and minor synapses with different gain factors. Such a method can be widely used in RRAM-based analog neuromorphic systems to increase the number of equivalent intermediate states exponentially. The proposed method also generates a more suitable Δ***W***, diminishing the distortion caused by sign SGD. Unlike when several RRAM cells are combined to achieve higher resolution, there are no carry operations for weighted synapses, even if a saturation on the minor synapses occurs. The proposed method also simplifies the circuit overhead, rendering it highly suitable to the parallel updating scheme. With the aid of weighted synapses, convergence is highly optimized, and the error rate decreases significantly. Weighted synapses are also robust against the intrinsic variations of RRAM devices.

## Introduction

Deep learning has made significant advances in many areas, such as image/speech recognition and natural language processing (LeCun et al., 2015). Currently, a powerful deep neural network often means huge networks scaled together with massive training data, which demands a learning system with excellent computational efficiency. However, conventional computer systems suffer from the von Neumann bottleneck between separate data processing (CPUs and GPUs) and data storage (memory). Thus, alternative technologies beyond the von Neumann bottleneck are attracting increased attention.

One emerging solution is resistive random access memory (RRAM)-based neuromorphic computing. Numerous studies have used RRAM-based neuromorphic systems as inference accelerators for neural networks (Li et al., 2015; Wang et al., 2015; Chi et al., 2016). RRAM-based neuromorphic systems can implement efficient matrix-vector multiplication in a large-scale crossbar array (Chi et al., 2016). Recently, some researchers discovered that RRAM-based analog neuromorphic systems can also dramatically accelerate the training of neural networks by carrying out certain parallel update schemes (Burr et al., 2015; Kataeva et al., 2015; Gokmen and Vlasov, 2016; Fuller et al., 2017; Gokmen et al., 2017). For a fully connected neural network layer that maps N neurons to M neurons, the weights updating time complexity of carrying out parallel update schemes is much lower than the usual time complexity of *O*(*N* × *M*). Parallel update schemes are generally based on stochastic gradient descent (SGD) (Bottou, 2010) or mini-batch gradient descent (GD) (more details will be discussed in section Online Learning). For SGD, the Δ*W* can be directly factorized to the multiplication of column and row vectors, which is ideally suited to *O*(1) parallel update schemes. For mini-batch GD, the time complexity of the corresponding update schemes is *O*(*mb*), where *mb* is the size of the mini-batch (Gokmen et al., 2017). Additional extra storage is required, however, for mini-batch GD.

Some researchers (Burr et al., 2015; Kataeva et al., 2015; Fuller et al., 2017) proposed one type of parallel update scheme based on variable-amplitude operation voltage *V*_*op*_. However, for some types of RRAM devices, multiple studies have demonstrated that the change in conductance at every step (*G*) is very sensitive to *V*_*op*_ (Woo et al., 2016; Wu et al., 2017), and it is very difficult to find a compact relation between them, such as linear or exponential. It is also challenging to design a variable-amplitude training scheme for such types of RRAM devices. Gokmen et al. (Gokmen and Vlasov, 2016) proposed another type of parallel update scheme based on stochastic computing. Their method, however, requires an extra circuit module to generate stochastic bit streams.

A simplified parallel update scheme based on sign SGD is a sensible way to reduce the hardware overhead of Δ-weights computation and bit stream generation. Thus, the magnitude of Δ*W* is ignored, and only its sign information is relevant. However, this type of scheme decreases accuracy. For SGD, ∇*J*(*t*) will be quite small when *J* (cost function) approaches its local minimum, ensuring that the algorithm converges even if the learning rate is constant. However, this scheme fails when the gradient vector's magnitude information is ignored. Considering the limited number of intermediate states (Woo et al., 2016; Fuller et al., 2017; Wu et al., 2017) and intrinsic variations of RRAM devices (Wong et al., 2012), the problem is even greater.

For these reasons, it is challenging to achieve an RRAM-based analog system with high performance. In this paper, we propose a new method to optimize the simplified parallel update scheme based on weighted synapses. The idea of combining several RRAM cells to achieve higher resolution in a digital system has been proposed by many studies (Chi et al., 2016; Song et al., 2017). For PipeLayer (Song et al., 2017), 16-bit weights are stored in four groups of RRAM cells. Each group stores 4-bit weights for the 15-12, 11-8, 7-4, and 3-0 segments, respectively. A carry operation occurs when a saturation on the less significant RRAM cell is encountered. The weighted synapses method differs, however, because weighted synapses are relatively equal, which means that there will be no carry operations even if a saturation on the minor synapse is encountered. There are two significant advantages of avoiding carry operations. The first advantage is speed. Carrying out the read operation row by row on an *N* × *N* RRAM-based crossbar array is inefficient with time complexity *O*(*N*). It does not match the parallel update scheme, whose time complexity is *O*(1). The other advantage is circuit overhead. Without carry operations, there are no extra control logic and ADC (analog to digital converter) circuits (which are designed to read the resistance of a certain RRAM cell) because there is no need to set the accurate resistance for a certain RRAM device.

With the assistance of weighted synapses, algorithm convergence is greatly optimized, and the error rate is significantly decreased for the task of recognizing handwritten digits trained on the Modified National Institute of Standards and Technology (MNIST) database with a two-layer perceptron, even if the number of intermediate states is small or the variation is large. The proposed method makes it much simpler for RRAM devices to meet the requirements of an analog neuromorphic system.

## RRAM-based neuromorphic system

This section provides background information about electronic synapses and RRAM technology and demonstrates a multilayer neuromorphic architecture.

### RRAM-based synapses

Electronic synapses were first made by circuits with complementary metal-oxide-semiconductor (CMOS) transistors and capacitors (Indiveri et al., 2006). Recently, electronic synapses based on memristor (Chua, 1971; Strukov et al., 2008) have received significant attention for their high density and extreme low-power potential (Yu et al., 2011; Burr et al., 2015; Kataeva et al., 2015; Li et al., 2015; Prezioso et al., 2015; Sheri et al., 2015; Soudry et al., 2015; Wang et al., 2015; Chi et al., 2016; Eryilmaz et al., 2016; Gokmen and Vlasov, 2016; Fuller et al., 2017; Gokmen et al., 2017; Yao et al., 2017). Significant progress has been made on applying such synapses to neural networks, such as Multilayer Perceptron (MLP) (Burr et al., 2015; Kataeva et al., 2015; Prezioso et al., 2015; Soudry et al., 2015; Fuller et al., 2017; Yao et al., 2017), Restricted Boltzmann Machine (RBM) (Sheri et al., 2015; Eryilmaz et al., 2016), and Convolutional Neural Network (CNN) (Gokmen et al., 2017; Song et al., 2017). For neuromorphic system learning, researchers have proposed different types of parallel update schemes (Burr et al., 2015; Kataeva et al., 2015; Gokmen and Vlasov, 2016; Fuller et al., 2017; Gokmen et al., 2017). Efforts have also been made to address the problems caused by high-precision computation and storage, which are difficult to realize in neuromorphic hardware (Neftci et al., 2017).

RRAM could also be treated as one type of memristor. In general, the basic structure of RRAM is a switching medium sandwiched between two electrodes. Nonvolatile storage is based on resistive switching between a low-resistance state (LRS or ON state) and a high-resistance state (HRS or OFF state) under voltage or current stimulation.

Recently, significant progress has been made in the performance of analog RRAM-based synapses (Woo et al., 2016; Fuller et al., 2017; Wu et al., 2017). Rather than utilizing the conventional multilevel characteristics of RRAM to construct a digital system (Chi et al., 2016; Song et al., 2017), attempts have been made to use analog synapses to achieve adaptive learning. The greatest benefit of an analog synapse is to change the conductance gradually, which is called its analog behavior. This process is achieved by applying a number of training pulses with equal intensity, rather than by varying the pulses' amplitude (Woo et al., 2016; Wu et al., 2017).

Ideally, the conductance of an analog synapse will increase by a fixed value (*G*) after applying a SET pulse and will decrease by the same value after applying a RESET pulse. However, *G* of an RRAM-based analog synapse device varies randomly after applying a certain training pulse due to device-to-device and cycle-to-cycle resistance switching variations (Wong et al., 2012). In addition, the number of intermediate states of an RRAM device is limited. According to state-of-the-art studies, the quantity of intermediate states is in the order of 50~200 (Woo et al., 2016; Fuller et al., 2017; Wu et al., 2017), which brings additional challenges to RRAM-based analog neuromorphic systems (Gokmen and Vlasov, 2016).

### Neuromorphic architecture

**Figure 1:**
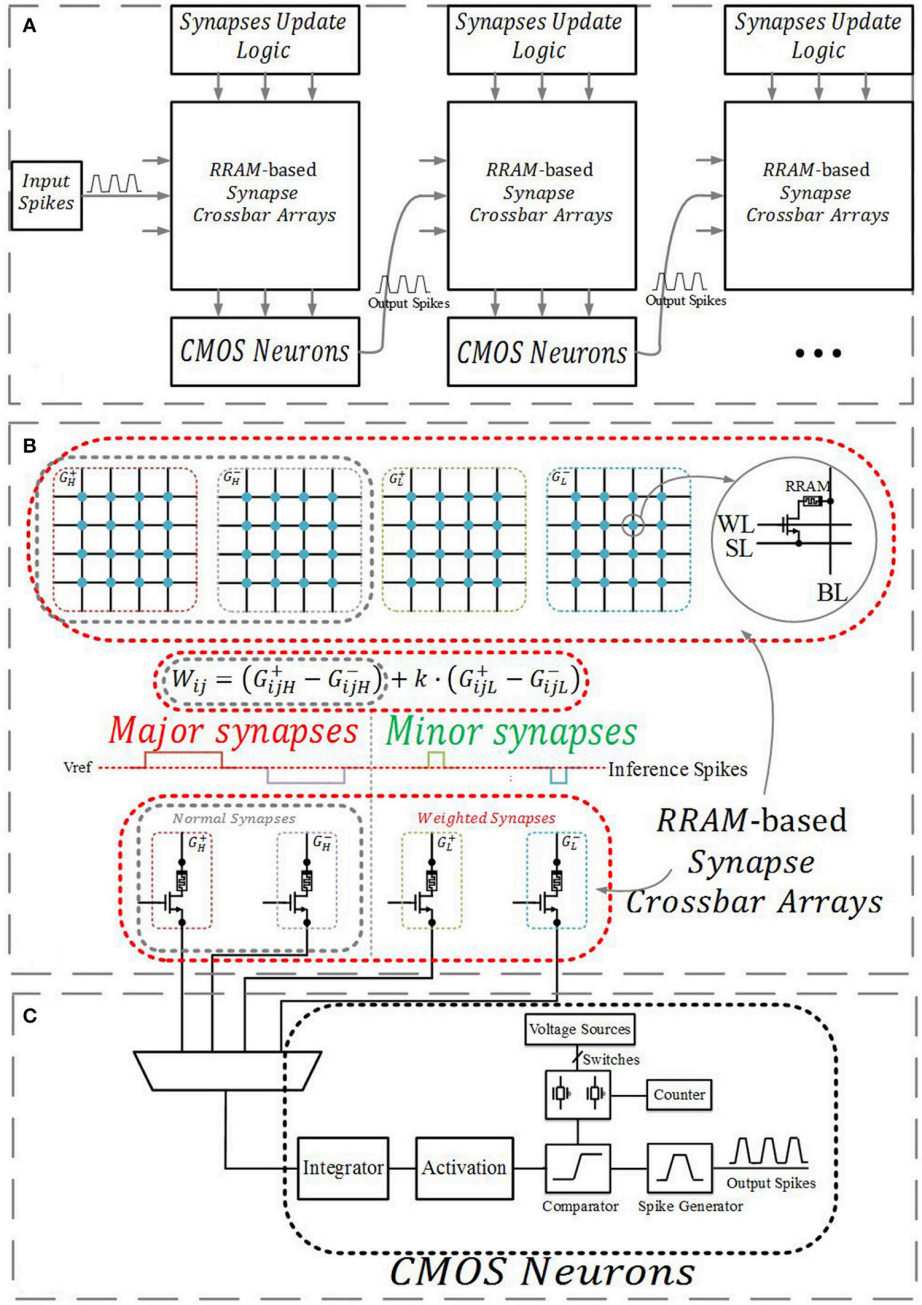
**(A)**The multilayer neuromorphic architecture. **(B)** RRAM-based synapses crossbar arrays. A normal synapse is implemented with two differential RRAM devices, and a weighted synapse is implemented with four RRAM devices. **(C)** CMOS neurons.

Figure 1 demonstrates a multilayer neuromorphic architecture, which consists of RRAM-based synapse crossbar arrays, CMOS neurons, and a synapse update logic circuit. It is built on a previous RRAM-based neuromorphic system (Kataeva et al., 2015; Fuller et al., 2017).

RRAM-based synapses connect the neurons of adjacent layers, and their conductance represents the weights in a neural network. Previous studies (Li et al., 2015; Wang et al., 2015; Chi et al., 2016) have shown that the matrix-vector multiplication in neural networks can be implemented efficiently in a crossbar structure as follows:

(1) $$\begin{array}{l} I_{i}=\sum W_{ij}V_{j}, \end{array}$$

where *V*_*j*_ consists of the input signals and a constant bias, *W*_*ij*_ are the adjustable synaptic weights, and *I*_*i*_ serves as the neuron's original output. As shown in Figure 1B, a normal synapse consists of two RRAM devices, and the value is given by $W_{ij}=G_{ij}^{+}-G_{ij}^{-}$, where $G_{ij}^{+}$ and $G_{ij}^{-}$ are the conductance of the corresponding differential RRAM devices. In our neuromorphic system, inference pulses serve as *V*_*j*_, including positive and negative pulses. Matrix-vector products *I*_*i*_ are integrated by an integrator, changing the potential of the integral capacity. The potential is conversed by a specific circuit, which serves as the activation function. Next, it is compared to a set of voltage sources, which manage the inference pulses fired out by the pulse generator, as shown in Figure 1C.

A weighted synapse consists of four RRAM devices, and their value is represented by $W_{ij}=\left(G_{ijH}^{+}-G_{ijH}^{-}\right)+k\cdot \left(G_{ijL}^{+}-G_{ijL}^{-}\right)$. Here $\left(G_{ijH}^{+}-G_{ijH}^{-}\right)$ is the conductance of the high significant synapse (major synapse), $\left(G_{ijL}^{+}-G_{ijL}^{-}\right)$ is the conductance of the low significant synapse (minor synapse), and *k* is a scale factor between 0 and 1 related to the relative width and voltage of inference pulses applied to the major and minor synapses. For example, if the width of inference pulses applied to the major synapses is 10 ns and the width of inference pulses applied to the minor synapses with the same voltage is 2 ns, the integrator will integrate 5 times the charge from the major synapses ($Q=\frac{V}{R}\times t$), so *k* = 0.2. Currently, a value of 15 on the major synapses means 15 and a value of 15 on the minor synapses means 15 × 0.2 = 3.

Synapses update logic takes charge of executing the simplified parallel update scheme, including calculating the error and derivative. More details will be discussed in section Online Learning.

## Online learning

This section demonstrates the computational process of the customized backpropagation (BP) algorithm based on sign SGD. Then it describes the updating logic of the proposed parallel update scheme. Finally, it demonstrates a two-layer perceptron (784 × 200 × 10) for the task of handwritten digit recognition trained on the MNIST database and analyzes the effects of some non-ideal factors of RRAM devices. Poor convergence and large error rate were observed, especially when the number of intermediate states was small.

### Customized BP algorithm

Error BP algorithms (Schiffmann et al., 1992; Neftci et al., 2017), the most successful learning method, are widely used in many types of neural networks, such as MLP, Recurrent Neural Networks (RNNs), and CNNs. However, there is no obvious advantage in utilizing the multilevel characteristics of an RRAM synapse in a digital system for implementing a BP algorithm. It is not easy to set the resistance of a certain RRAM cell accurately; it requires a large number of program operations and highly accurate circuit design.

As mentioned in section Introduction, we performed a simplified parallel update scheme based on sign SGD. Its time complexity was *O*(1), and its updating logic circuits were simple. The SGD method is an iterative procedure for obtaining the parameter values that minimize a function. As applied in a BP algorithm, the weights of a neural network serve as the parameters of the error function; at each training iteration, their values are modified in the direction where the error function decreases most rapidly (Rumelhart et al., 1988). Geometrically, the error function specifies an error surface defined over weight space. The procedure can be described as follows:

(2) $$\begin{array}{l} W\left(t+1\right)=W\left(t\right)-\eta \cdot \nabla J\left(t\right), \end{array}$$

where *W*(*t*) is the matrix of weights at time *t*, η is a constant learning rate, and *J*(*t*) is the cost function to be minimized.

**Figure 2:**
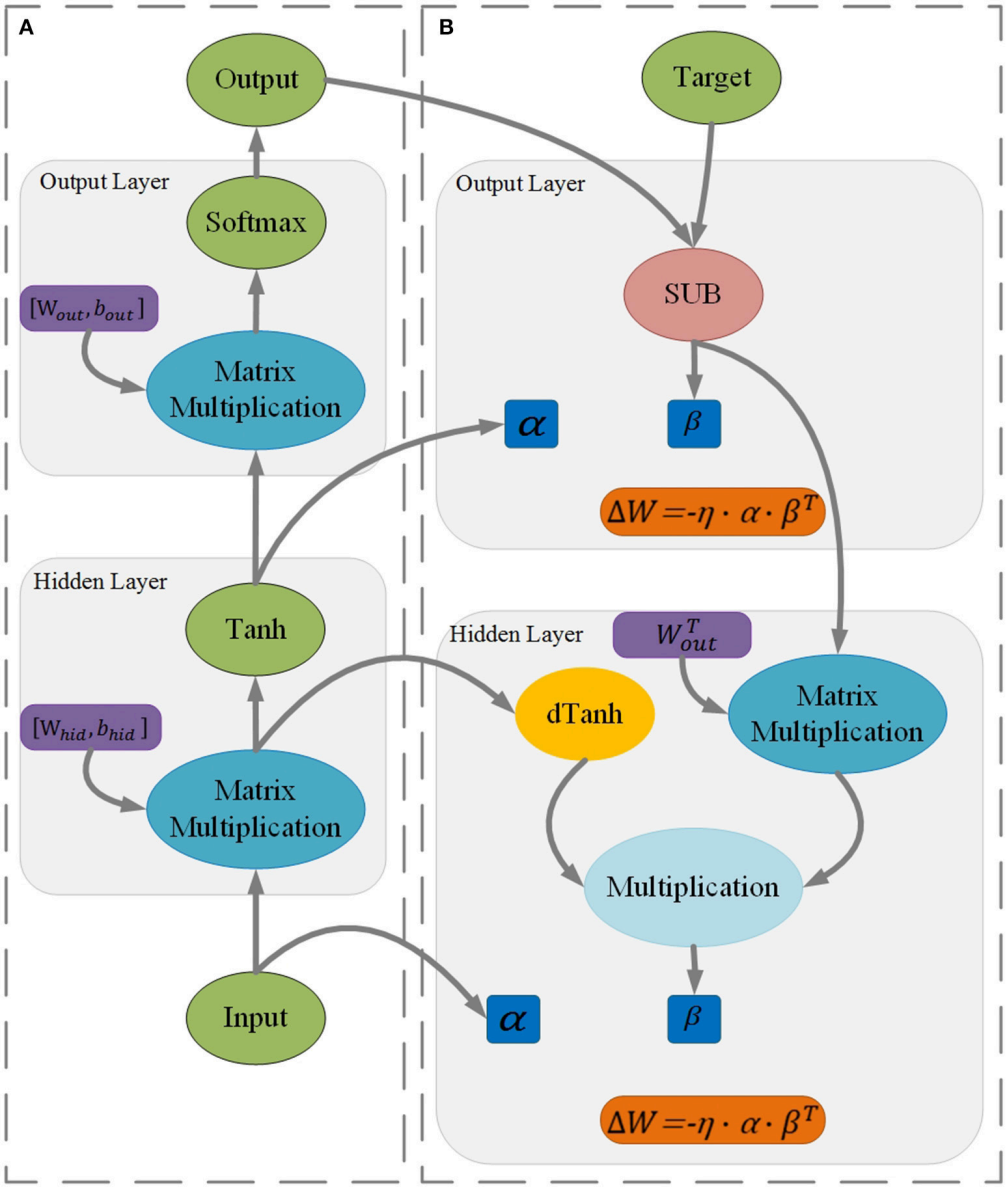
Procedures of inference **(A)** and learning **(B)** for a two-layer perceptron (Rumelhart et al., 1988).

Figure 2A shows the inference of a two-layer perceptron. The activation function is a hyperbolic tangent, and the cost function is the cross entropy. The output of the hidden layer is given by the following:

(3) $$\begin{array}{l} \alpha_{output}=\text{tanh}\left(W_{hidden}\times \alpha_{hidden}\right) \end{array}$$

The output of the output layer is given by the following:

(4) $$\begin{array}{l} \text{y}=\text{softmax}\left(W_{output}\times \alpha_{output}\right), \end{array}$$

where α is the input vector and *W* is the weight matrix of the corresponding layer.

Figure 2B shows the training of a two-layer perceptron. β is the error vector backpropagated from the next layer. The error vector of the output layer is given by the following:

(5) $$\begin{array}{l} \beta_{output}=y-y_{target}, \end{array}$$

where *y*_*target*_ is the desired output of the output layer. The error vector of the hidden layer is given by the following:

(6) $$\begin{array}{l} \beta_{hidden}=dtanh\left(W_{hidden}\times \alpha_{hidden}\right)\cdot \left(W_{output}^{T}\times \beta_{output}\right), \end{array}$$

where *dtanh* is the derivative of the activation function.

For SGD, the increment of the weights matrix is calculated by the following:

(7) $$\begin{array}{l} \Delta W=-\eta \cdot \alpha \cdot \beta^{T} \end{array}$$

The key concept of sign SGD is its focus on the sign information of Δ*W* rather than its magnitude. Thus, Equation (7) is translated to the following:

(8) $$\begin{array}{l} \Delta W=-{\Delta W}_{m}\cdot \alpha^{\prime }\cdot {\beta^{\prime }}^{T}, \end{array}$$

where α′ = sign(α) and β′ = sign(β) represent the sign information of α and β and Δ*w*_*m*_ is determined by the number of intermediate states of the corresponding RRAM devices. Obviously, Δ*w*_*m*_ cannot be arbitrarily small because the number of intermediate states of RRAM devices is limited.

### Updating logic of the parallel update scheme

**Figure 3:**
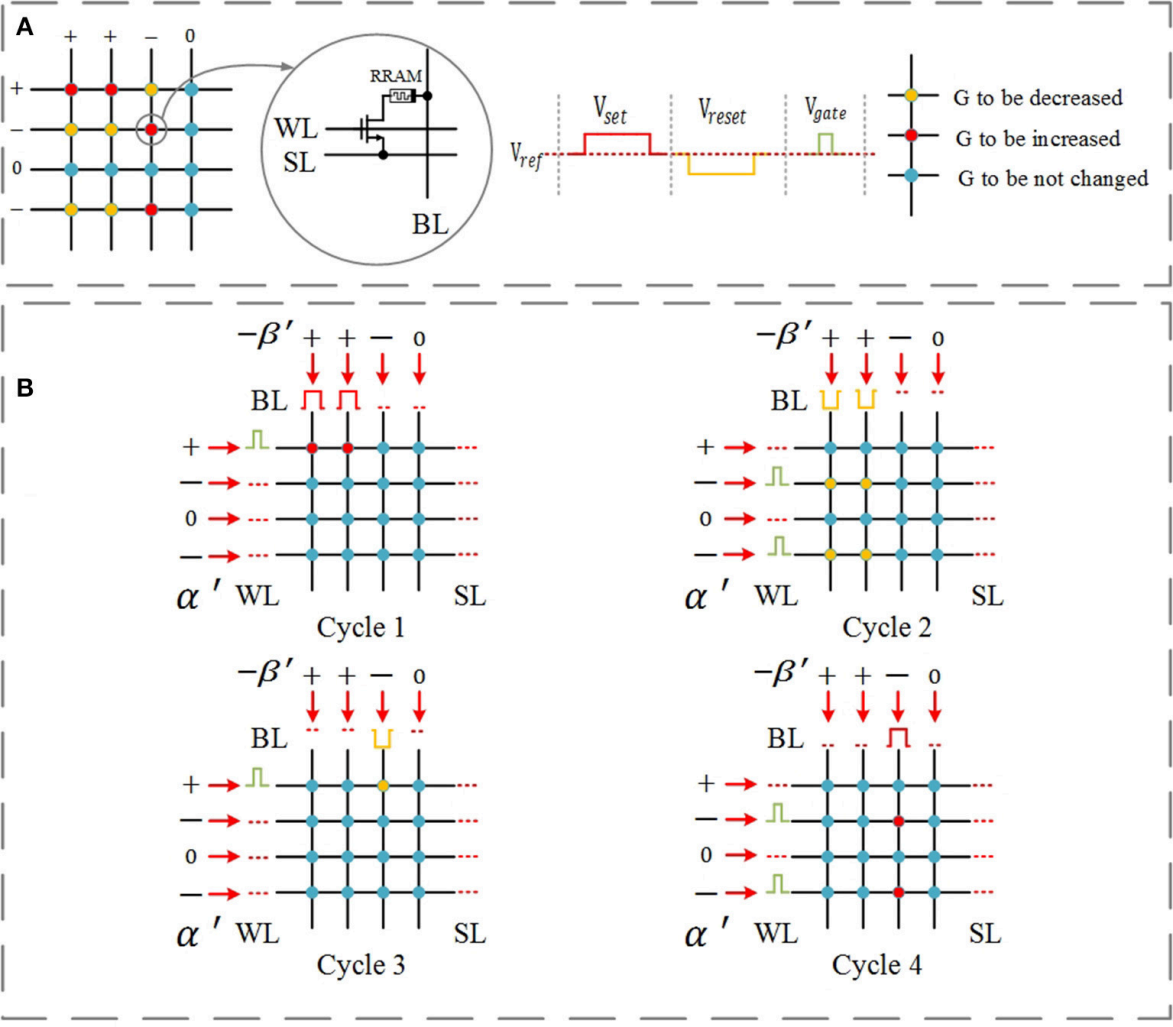
Simplified parallel update scheme for a single layer with four inputs and four outputs. The update scheme for the corresponding differential crossbar array is just the opposite, and it is carried out simultaneously. **(A)** Considered example (left) and the waveform of operation pulses (right). The red cells need a SET operation, and the yellow cells need a RESET operation. **(B)** Cycle 1–SET pulses are applied to adjust the devices whose corresponding inputs α′ = +1 and error β′ = −1 (red); Cycle 2–RESET pulses are applied to adjust the devices whose corresponding inputs α′ = −1 and error β′ = −1 (yellow); Cycle 3–RESET pulses are applied to adjust the devices whose corresponding inputs α′ = +1 and error β′ = +1 (yellow); and Cycle 4–SET pulses are applied to adjust the devices whose corresponding inputs α′ = −1 and error β′ = +1 (red).

Figure 3 demonstrates an example of executing the simplified parallel update scheme. α′ and β′ represent the sign information; they can only consist of +1, −1, and 0. The bias pulses applied on word lines depend on the value of α′, and the bias pulses applied on bit lines (BLs) depend on the value of −β′. Figure 3A shows the given example (left) and the waveform of the programming pulses (right). The red cells need a SET operation, and the yellow cells need a RESET operation.

The updating scheme is carried out in 4 cycles, as shown in Figure 3B: Cycle 1–SET pulses are applied to adjust the devices whose corresponding inputs α′ = +1 and error β′ = −1 (red); Cycle 2 –RESET pulses are applied to adjust the devices whose corresponding inputs α′ = −1 and error β′ = −1 (yellow); Cycle 3–RESET pulses are applied to adjust the devices whose corresponding inputs α′ = +1 and error β′ = +1 (yellow); Cycle 4–SET pulses are applied to adjust the devices whose corresponding inputs α′ = −1 and error β′ = +1 (red).

### Simulation results and analyses

**Figure 4:**
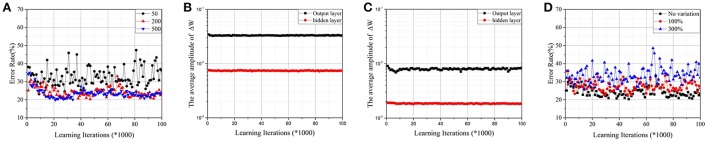
**(A)**Test error on the MNIST dataset by the parallel update scheme with normal synapses. The RRAM devices have 50, 200, and 500 intermediate states, respectively. **(B)** The average amplitudes of **ΔW** against learning iterations for RRAM devices with 50 intermediate states. **(C)** The average amplitudes of **ΔW** against learning iterations for RRAM devices with 200 intermediate states. **(D)** The effect of variation for RRAM devices with 200 intermediate states. The ratios of the standard deviation and mean value are 0% (no variation), 100%, and 300%, respectively.

A two-layer perceptron (784 × 200 × 10) trained on the MNIST database for the task of recognizing handwritten digits was demonstrated. The MNIST training set was split into two parts—one with 50,000 images was used for training, and another with 10,000 images was used for validation. For convenience, the corresponding *W*_*ij*_ of a synapse was scaled to [−1, 1], and the initial value was chosen randomly. It was assumed that ${\Delta w}_{m}=\frac{1}{n}$ and *n* was the number of intermediate states. As shown in Figure 4A, when the number of intermediate states was low, convergence was poor. It is a commonly known problem that if the learning rate is too large, the cost function *J* may not decrease on every iteration, and SGD may never converge. Due to the limited quantity of intermediate states of RRAM devices, Δ*w*_*m*_ will be quite large and cause a similar problem. Accuracy is apparently lost, however, when the number of intermediate states increases to 500 due to sign SGD. For SGD, ∇*J*(*t*) will be relatively small when *J* approaches its local minimum, ensuring that the algorithm converges even if the learning rate is constant. However, it fails when the gradient's magnitude information is ignored. As shown in Figures 4B,C, the average amplitudes of Δ*W* do not decrease as learning iterations increase, meaning that ∇*J*(*t*) does not decrease even if *J* approaches its local minimum. The result is accuracy loss.

Figure 4D demonstrates the effects of variation for RRAM devices with 200 intermediate states; the results are similar for RRAM devices with 50 or 500 intermediate states. To develop a compact RRAM model with variation, we assumed that Δ*G* is subject to Gauss distribution (so is Δ*w*_*m*_). The variation can be evaluated by the ratio of the standard deviation and the mean value. The results show that large variation also damages the performance of the parallel update scheme.

## Weighted synapses

This section proposes a new method based on weighted synapses and explains why it optimizes the convergence of the simplified parallel update scheme. With the help of weighed synapses, algorithm convergence is greatly optimized, and the error rate decreases significantly, even if the number of intermediate states is small. Weighted synapses are also robust against the variation of RRAM devices.

### Optimization with weighted synapses

As analyzed above, the limited number of intermediate states of RRAM devices causes a problem similar to the too-large learning rate. Sign SGD brings apparent accuracy loss. Therefore, these parallel update schemes have at least two major issues: (1) it is challenging to generate a small enough Δ*w*_*m*_ due to the properties of RRAM devices; and (2) too much valid information is ignored by sign SGD. A 1-bit SGD (Seide et al., 2014; Wen et al., 2017) is a potential solution to the second issue, although it requires extra control logic circuits and storage. The problem caused by the limited quantity of intermediate states, however, still exists.

To optimize an *O*(1) update scheme, we propose a novel method based on weighted synapses. It can also be applied in 1-bit SGD after slight modifications.

As mentioned in section RRAM-based Neuromorphic System, a weighted synapse consists of four RRAM devices, and the value is given by $W_{ij}=\left(G_{ijH}^{+}-G_{ijH}^{-}\right)+k\cdot \left(G_{ijL}^{+}-G_{ijL}^{-}\right)$. With weighted synapses, there are only slight changes in the calculation of Δ*W*. Now Δ*W* is also calculated as Equation (7), but β′ is no longer sign (β). Only when the error vector from backpropagation (β) is greater than a given threshold (*T*) will its corresponding major synapse be updated. When β is greater than *k* · *T*, its corresponding minor synapse will be updated. Otherwise, there will be no updating procedure.

Thus, a smaller Δ*w*_*m*_ can be achieved with the same quantity of intermediate states. Moreover, ∇*J*(*t*) decreases as *J* (cost function) approaches its local minimum because there is no updating procedure if the error vector from backpropagation (β) is smaller than *k* · *T*.

### Choosing proper parameters (*k,T*)

A proper threshold (*T*) can be chosen by testing some values (such as 0.3, 0.1, 0.03, etc.), similar to choosing a learning rate. In practice, we found that a growing threshold results in better performance, but it also causes more difficulties in circuit design. For a two-layer perceptron, a fixed threshold is effective.

**Figure 5:**
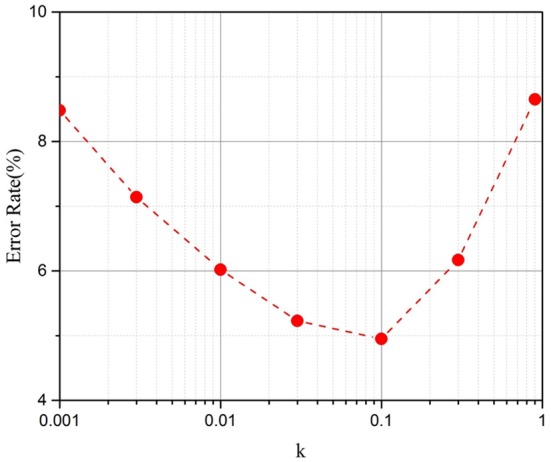
Test error on the MNIST dataset after 100,000 training iterations with different *k* (Taking the RRAM devices with 50 intermediate states as an example).

The simulation results for different *k* values are demonstrated in Figure 5. The results show that the method works well with a *k* between 0.03 and 0.1. For later simulations, we chose *k* = 0.1. When *k* is equal to 0 or 1, there is no updating procedure if the error vector from backpropagation (β) is smaller than *T*. Its updating frequency will be lower than the original parallel update scheme; thus, its performance is comparatively better.

### Simulation results

**Figure 6:**
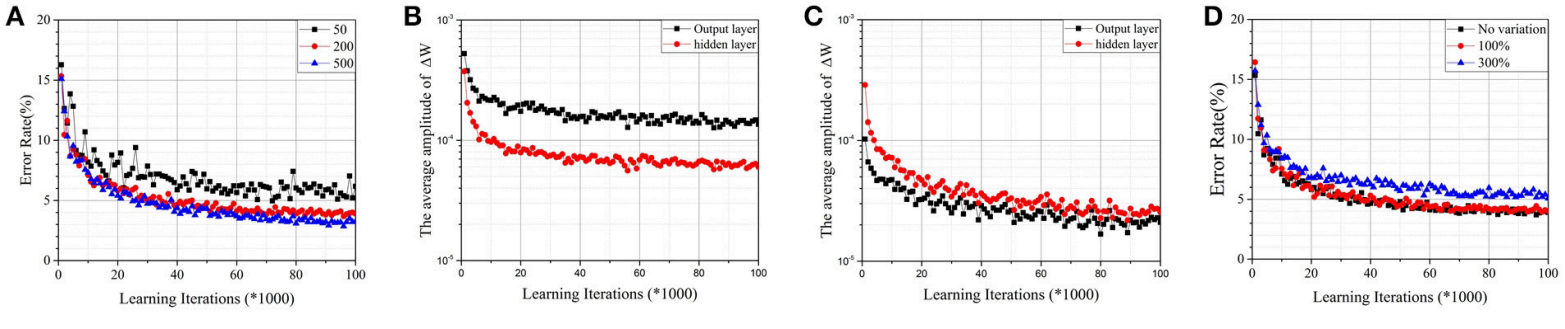
**(A)** Test error on the MNIST dataset by the parallel update scheme with weighted synapses (*k* = 0.1). The RRAM devices have 50, 200, and 500 intermediate states, respectively. **(B)** The average amplitudes of **ΔW** against learning iterations for RRAM devices with 50 intermediate states. **(C)** The average amplitudes of **ΔW** against learning iterations for RRAM devices with 200 intermediate states. **(D)** The effect of variation for RRAM devices with 200 intermediate states. The ratios of the standard deviation and mean value are 0% (no variation), 100%, and 300%, respectively.

Figure 6A shows the test error with weighted synapses. The convergence is greatly optimized, and the error rate decreases significantly, even if the number of intermediate states is small. For RRAM devices with 50 intermediate states, the error rate decreases more than fivefold (obtaining 4.9%). With more intermediate states, the error rate declines further. For RRAM devices with 200 intermediate states, an error rate around 3% can be achieved in 100,000 learning iterations (2 epochs of the training set). With further learning iterations and a growing threshold (*T*), the error rate can be reduced to around 2.3%, which is comparable with the performance of other RRAM-based systems (Burr et al., 2015; Kataeva et al., 2015; Gokmen and Vlasov, 2016; Fuller et al., 2017). Comparing with the average amplitudes of Δ*W* in Figures 4B,C, the corresponding values in Figures 6B,C are much smaller. Moreover, the values are apparently decaying as the learning iterations increase, guaranteeing that the equivalent ∇*J*(*t*) is quite small when *J* approaches its local minimum. Both result in significantly improved accuracy.

Figure 6D demonstrates the effect of variation for RRAM devices with 200 intermediate states; the results are similar for RRAM devices with 50 or 500 intermediate states. Thus, weighted synapses are robust against variation.

## Conclusion

This paper proposes a new method based on weighted synapses to optimize the parallel updating scheme in RRAM-based neural networks. It helps address some critical issues of implementing a parallel update scheme, such as the limited number of intermediate states of RRAM devices and the substantial gradient distortion. The proposed optimization method is also robust against variation. For example, in RRAM devices with 50 intermediate states, the error rate decreases more than fivefold (obtaining 4.9%).

## Author contributions

YL proposed the method based on weighted synapses and performed most analyses. HW and BG checked its feasibility in RRAM-based neuromorphic system. ND and HQ conducted the studies about RRAM devices. QZ performed some analyses on algorithms. YL, HW, and BG prepared the manuscript.

### Conflict of interest statement

The authors declare that the research was conducted in the absence of any commercial or financial relationships that could be construed as a potential conflict of interest.
